# Anticoagulation in patients with mechanical heart valves: follow the guidelines!

**DOI:** 10.1007/s12471-014-0642-9

**Published:** 2015-01-21

**Authors:** Freek W.A. Verheugt

**Affiliations:** P.C. Hooftstraat 188, 1071 CH Amsterdam, The Netherlands

Heart valve surgery has been one of the great leaps forward in the management of heart disease in the last 60 years. Thanks to technical developments, artificial heart valves were constructed that prove to be competent and durable. Besides the risk of infective endocarditis, thromboembolism from the foreign body structures of the prosthesis remains a major problem, which can be effectively reduced, but not annihilated by the use of oral anticoagulants. For nearly all patients on oral anticoagulants, bleeding is the most common problem. The only available and proven effective oral anticoagulants for carriers of mechanical heart valve prosthesis are the vitamin-K antagonists (VKA). They need intensive monitoring of the anticoagulation status through the dense network of thrombosis clinics in the Netherlands. Recently, non-VKA direct-acting oral anticoagulants were introduced, which are safer and more effective than VKA in stroke prevention in atrial fibrillation [1]. They have also been tested in patients undergoing mechanical heart valve replacement and in those with recent implantation, but here they failed in efficacy and safety in comparison with VKA [2]. Thus, VKA remains the standard of anticoagulation care for patients carrying artificial heart valves.

In the today’s issue of the *Netherlands Heart Journal* the results of a retrospective registry of mechanical aortic valve replacement (AVR) are presented by Swinkels et al. [3]. Data were collected in a single-centre experience and have a very long follow-up. Bleeding complications were collected in relation to age (< 60, 60–65 and > 65 years) at the time of surgery. The authors found a high rate of major bleeding per year: the lowest in the youngest age group and the highest in the oldest. Intracranial haemorrhage was seen at an unacceptable rate (0.6–0.7/year) in all age groups. Therefore, the authors conclude that the use of mechanical AVR below the age of 60 years is associated with a similar rate of intracranial bleeding as seen in older patients.

This report shows the deleterious effect of over-anticoagulation in many patients with a low-risk mechanical aortic prosthesis. Bleeding on VKA is highly dependent on INR monitoring. Since 2007 international guidelines recommend a target INR of 2.5 in carriers of a mechanical aortic valve without risk factors such as atrial fibrillation. The St. Jude Medical prosthesis in the aortic position, which was used in half of the patients, is considered a valve with a low thrombotic risk and only very few of the patients under 60 years of age had atrial fibrillation. Therefore, the bleeding—especially intracranial bleeding—encountered in this cohort is unacceptable. Of course, this knowledge was not available at the time the first patients were included in this cohort; however, already in 1996 the Leiden group published the low thrombosis rate in this type of valve [4]. And in 2006 and 2007 the American [5] and European [6] guidelines became available and have not changed on this topic since then (Fig. 1) [7]. But it was not until 2010 that the Netherlands Federation of Thrombosis Clinics mentioned, in their yearly report, the existence of a differentiated approach for patients with artificial heart valves [8]. This explains why the rate of major bleeding described in this cohort did not seem to drop over the years.

**Fig. 1:**
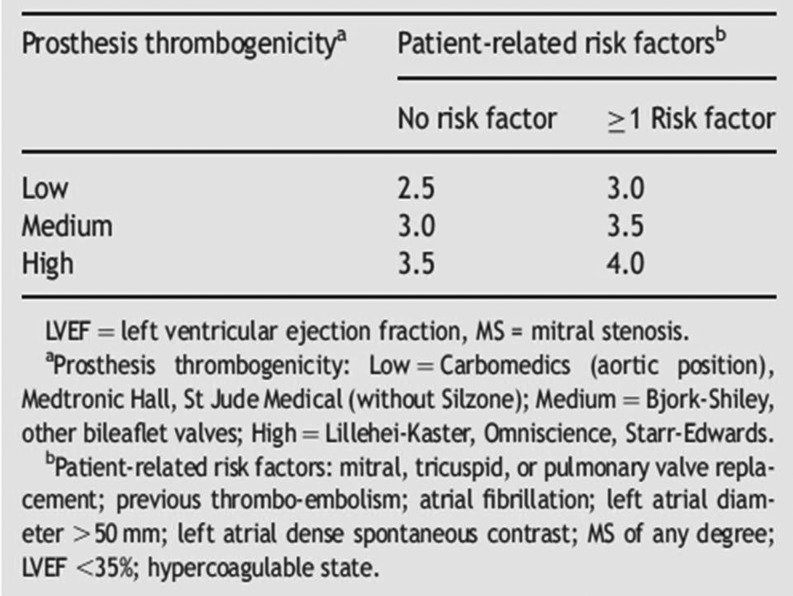
2007 recommendations of the European Society of Cardiology on the INR in the anticoagulant management of patients with mechanical heart valve prosthesis. [6]

Thus, knowledge of the guidelines is essential in the management of patients in general, but of this group of patients with a delicate balance between thrombosis and bleeding in particular.

## References

[1] Verheugt FWA (2013). The new oral anticoagulants in atrial fibrillation: an update. Neth Heart J.

[2] Eikelboom JW, Connolly SJ, Brueckmann M (2013). Dabigatran versus warfarin in patients with mechanical heart valves. N Engl J Med.

[3] Swinkels BM, De Mol BA, Kelder JC, Vermeulen FE, Ten Berg JM. Long-term bleeding events after mechanical aortic valve replacement in patients under the age of 60. Neth Heart J. 2014. doi.10.1007/s12471-014-0626-9. 10.1007/s12471-014-0626-9 PMC4315789 25408510

[4] Cannegieter SC, Rosendaal FR, Wintzen AR, Van der Meer FJ, Vandenbroucke JP, Briet E. (1995). Optimal oral anticoagulant therapy in patients with mechanical heart valves. N Engl J Med.

[5] Bonow RO, Carabello BA, Chatterjee K (2006). ACC/AHA 2006 guidelines for the management of patients with valvular heart disease: a report of the American College of Cardiology/American Heart Association Task Force on Practice Guidelines. Circulation.

[6] Vahanian A, Baumgartner H, Bax JJ (2007). Guidelines on the management of valvular heart disease. Eur Heart J.

[7] Vahanian A, Alfieri O, Andreotti F (2012). Guidelines on the management of valvular heart disease. Eur Heart J.

[8] http://www.fnt.nl/algemeen/jaarverslag_FNT_medisch_2010.pdf

